# Analysis of non-squamous vulvar cancer cases: A 21-year experience in a single center

**DOI:** 10.4274/tjod.83436

**Published:** 2014-09-15

**Authors:** Derya Akdağ Cırık, Rukiye Kalyoncu, Işın Üreyen, Tolga Taşçı, Nurettin Boran, Ahmet Özfuttu, Taner Turan, Gökhan Tulunay

**Affiliations:** 1 Ankara Etlik Zübeyde Hanım Women’s Health Education and Research Hospital, Ankara, Turkey

**Keywords:** Non-squamous vulvar cancer, malignant melanoma, basal cell carcinoma

## Abstract

**Objective:**

To evaluate the patients with non-squamous cell type of vulvar cancer who were treated in our clinic within 21 years.

**Materials and Methods:**

We assessed the data of 14 patients who were treated for non-squamous cancer of the vulva between January 1992 and August 2013. The age of patients, histopathological diagnosis of the tumor, tumor size, tumor location, medical or surgical treatment, response to the treatment, recurrence, and survival rates were analyzed.

**Results:**

The mean age of the patients was 53 years. The main complaint was vulvar pruritus (71%). Mean tumor size was 2.4 cm (range: 0.5-6 cm). In 65% of cases, the tumor was localized in the labia majora. The histopathologic diagnosis of the patients was as follows: malignant melanoma in 5 patients, basal cell carcinoma in 5 patients, mucinous type adenocarcinoma in 2 patients, apocrine gland carcinoma in one patients, and malign peripheral nerve sheath tumor in 1 patient. For 11 patients, surgery was the primary treatment. Radical vulvectomy and bilateral inguinofemoral lymphadenectomy were performed in 8 patients. Local excision alone without lymphadenectomy was performed in other 3 patients. Five of eight patients (62.5%), who undergone radical surgery, had lymph node metastases. Of these 5 patients, two had bilateral lymph node metastasis. Mean follow-up time was 49.2 months (range 12 to 72 months). Eight (57.1%) patients had suffered first recurrence. In those patients, the mean time to recurrence was 19.5 months (range, 6-48 months).

**Conclusion:**

Non-squamous cell vulvar cancer is a rare disease and comprises a heterogeneous group of tumors. Malignant melanoma is the most aggressive one. Multicenter prospective studies are necessary in order to standardize the treatment of these rare tumors.

## INTRODUCTION

Vulvar cancer is a rare disease and it accounts for 0.3% of all cancers in women and 5%-6% of genital tract cancers. Squamous cell vulvar cancer is the most common histological type of vulvar cancer and it is responsible for 85%-90% of all cases^(1)^. On the other hand, malign melanoma (MM) is the most common histological type within non-squamous vulvar cancers and it accounts 5% to 10% of the vulvar cancers. The other histologic types are as following:vulvar sarcoma (1%-2%) and basal cell cancer (2%), bartholin’s gland carcinoma, adenocarcinoma and undifferentiated vulvar cancers^(2)^.

Although vulvar cancer is more frequently diagnosed in hypertensive, diabetic, obese, heavy smoker and chronic immunosuppressive women, the etiology is not fully understood^(3)^. Vulvar cancer is usually a post-menapousal disesaseandhas a slow clinical course. If it is diagnosed at an earlier stages, the treatmentis more succesfull. However, an effective method for early diagnosis has not introduced yet.

In this study, we aimed to analyze thepatients who were treated for non-squamous cell type vulvar cancer between January 1992 and August 2013 in our hospital.

## MATERIALS AND METHODS

The clinico-pathological datas of the women, referred to our hospital with a diagnosis of non-squamous cell type vulvar cancer, between January 1992 and August 2013 was retrospectively analyzed. A total of 136 patients with vulvar cancer were reviewed for the study. Non-squamous cell vulvar cancer were diagnosed in 14 patients and they eligible for the study.

All patients routinely examined for cervicovaginal smearand colposcopy if necessary. Chest radiograph, upper abdominal and pelvic ultrasonography, abdominal computerized tomography examinations were the other routine pre-operative radiologic examinations. For patients diagnosed with malignant melanoma, thoracoabdominal computerized tomography and if necessary cranial magnetic resonance imaging was also performed. Clinico-pathologic data of the patients were reviewed from patient’s files, and computerized database.

Tumor location was classified as ‘midline’ when the tuumor was within 2.5 cm distance from midline and ‘lateral’ when the tumor 2.5 cm far from the midline. Tumor size was calculatedby measuringthe largest diameter of the tumor. Histopathological data such as the number of resected lymp nodes, lymp node positivity, were reviewed from pathology records of the patients.

When lymph node or distant metastasis were detected after surgery, patients were given systemic chemotherapy (vincristine, bleomycin) or concomitant chemoradiotherapy, as an adjuvant therapy. Primary radiotherapy or concomitant chemoradiotherapy was given to patients with advanced disease and who were not undergone surgery due to additional diseases.

One month after adjuvant treatment, if no tumor was detected in clinical examination and/or imaging technics itwas defined as; ‘complete clinical response’; havingmore than 50% reductionintumorwas defined as; ‘partial clinical response’; shrinkage between 25%-50% of tumor was defined as; ‘stable disease’; and not less than 25% shrinkage or emergence of new lesion was defined as; ‘progressive disease’.

Patients were called for postoperative control three months intervals in the first year and 6-month intervals in subsequent years. Disease free survival was defined as the time period from diagnosis to recurrence. The overall survival was defined as; the time period from diagnosis to death.

## RESULTS

The average age of diagnosis was 53, ranging between 22-68 years. The most frequent complaint was vulvar pruritis (71%). The average tumor size was 2.4 cm, ranging from 0.5 to 6 cm. The most common location of the tumor was labia majora (65%). According to the histopathological results; 5 patients were diagnosed with MM, 5 with basal cell carcinoma, 2 withmucinous type adenocarcinoma, one with apocrine gland carcinoma and one with malignant peripheral nerve sheat tumor.

Five of the patients with non-squamous vulvar cancer (35.7%) were diagnosed as MM. Of those patients, tumor site was labia majora in two patients, clitoris in two and labia minora in one case. In these patients, the mean tumor diameter was 1.9 cm, ranging from 0.5 to 4 cm. The most frequent complaint was itching and discoloration of the vulva. Tumor characteristics of patients, the treatment and follow-up protocols were presented in (Table 1). Three of malignant melanoma cases were performed radical vulvectomy and bilateral inguinofemoral lymph node dissection as primary therapy. In two of these three cases, lymph node metastases were detected and adjuvant radiotherapy was given to one case with bilateral lymph node involvement (patient no #1). Other lymph node positive patient with MM was took adjuvan therapy according to medical oncology consultation and called for routine follow-up (patient no #12). Other two MM patients, had either had multiple metastases in the liver parenchymeor had ametastatic lymph node,measuring 10 cm in the right groin (patient no #11 and #13). Due to this reason, surgery were not appropriate for them; radiotherapy and concomitant chemoradiotherapy was given. Three of patients with MM undergoing radical surgery was achieved complete clinical response after treatment. In one case thatunderwentsurgery, disease progressionwas detected during therapy. Complete clinical response was obtained in the other one (patient no #13).

In all 5 cases of vulvar MM, recurrence developed during follow-up. In 3 patients who were treated with radical surgery, recurrence was developed at 12, 12 and 24 months after surgery. Recurrence localization was as following; the breast, cervix, pelvic bones, urethra and vagina. For breast recurrence surgery was the choice of treatment, for cervical recurrence chemoterapy was given, for vaginal recurrence radiotherapy was given and for urethral recurrence multi-drug chemotherapy (vincristine + bleomycin + cisplatin + methotrexate. BOMP) was given. In three recurrent cases who had undergone radical surgery, tumor were totally resected. The patient who could not be operated due to widespread metastasis in liver died 12 months after diagnosis. The other MM case who was given radiotherapy, died 9 months after diagnosis.

Five patients were diagnosed with basal cell cancer of the vulva. Tumor localization was labia majora in four of them and clitoris in one of them. The mean tumor size was 1.5 cm ranging from 1 to 4 cm. In two of the patients diagnosed with basal cell cancer, radical vulvectomy and bilateral lymp node dissection was performed, and for other three cases local tumor excision was performed. In one of the patients who underwent radical surgery, unilateral lymph node metastases was detected (patient no #4). In that case, recurrence occured in vagina 30 months after diagnosis and chemotherapy (BOMP) was given for treatment. There was complete response to treatmet for recurrence.

In one patient, the histological diagnosis was apocrine carcinoma (patient no #3). In this case, tumor was 4 cm in diameter and localized in labia majora. Radical surgery was doneand adjuvant chemoradiotherapy was given for this case. Bilateral lymph node metastases were detected and there was no recurrence in 62 months follow-up period.

Patient with a diagnosis of malignant peripheral nevre sheath tumor (patient no #5) was 22 years old, the tumor was 3 cm in diameterand localized in labia majora. Radical vulvectomy and bilateral inguinofemoral lymph node dissection was performed for this case. No lymph node metastasis was detected after lymphaedenectomy and no adjuvant therapy was given. There was no recurrence at 28 months follow-up in this case.

In two cases of mucinous adenocarcinoma, tumor localization was labia majora and labia minora, 5 cm and 6 cm in diameter, respectively. Radical vulvectomy and bilateral inguinofemoral lymph node dissection was performed for that cases. First case had unilateral lymph node involvement (one lymph node) and did not receive adjuvant therapy (patient no #14). Inguinal recurrence developed 48 months after diagnosis and surgical resection of recurrent tumor was done in that patients. This patient was disease-free during 66 months period of follow-up. In the other patient with mucinous adenocarcinoma, surgery was not appropriate treatment due to presence of tumoral spread to the upper 2/3 vagina. This patient was given primary radiotherapy and complete response was achieved. The second recurrence was occured in the bladder after 12 months following the first recurrence. This patient did not accept the treatment of second recurrence and died 25 months after diagnosis.

In our case series, inguinal lymph node metastasis was detected in 5 of 8 patients who were undergone radical surgery. Metastatic lymph node was located unilaterally in three patients and bilaterally in two patients. In one case with bilateral lymph node involvement, the tumor was located in midline and in lateral side in the other one. Lesion was located laterally in all three cases with unilateral lymph node involvement.

Mean follow-up time was 49.2 months ranging from 12 to 72 months. Recurrence developed in eight patients. The average time from diagnosis to recurrence was 19.5 months (6-48 months). The mean duration of hospitalization was 15 days (8-12 days). Surgical site infection and inguinal wound disruption was observed in two patients as early complications.

## DISCUSSION

Non-squamous vulva cancers consist of a spectrum of tumors including MM, basal cell cancer, bartholin gland cancer, sarcoma and lymphoma. These tumors extend from basal cell cancer, which can be easily treated with local excision to Merkel cell tumor which has a very poor prognosis^(4)^. Due to its rarity and presence of different clinico-pathologic characteristics for each, there is no standardized treatment regimen for these tumors. Depending on the tumor histology, surgical management and if needed adjuvant treatment required was determined^(5)^.

Contrary to squamous cell cancer, non-squamous vulvar cancer is not only diagnosed in postmenopausal women but also young women. In presented series, patients’ mean age was 53 rangingfrom 22 to 68 years, 78% of these patients were postmenopausal and 28% of them were over the age of sixty. Tumor is often localized in labia majora for tumors with non-squamous cell types, but clitoris is a special localization for MM^(5,6)^. In our study, tumor were localized in clitoris in 40% of MM cases. Similar to squamous cell vulvar cancer, the most common complaint islong-lasting vulvar itching^(6,7)^. In accordance with the literature, 71% of cases in this series admitted to hospital with vulvar itching.

Because of the rarity of the malign melanomas of the genital tract, there were nostudy with large number of patients in the literature. Therefore there is no consensus about treatment modalities and follow-up schedules of these cases. For vulvar melanoma which has very poor prognosis, surgical treatment is shaped according to the information about current gynecological cancers and cutaneous melanom^(8)^. Localized disease, negative lymph node involvement, young age are good prognostic factors for survival. The metastasis of inguinofemoral lymph node is positively correlated with the depth of tumour invasion defined for malign melanoma in the Breslow microstaging system. For MM, studies show that there is no significant survival advantage of radical surgery compared to conservative approach, if the depth of invasion is less than 1mm. Solocal excision may also be a choice of treatment for such cases^(9)^. But, elective lymph node dissection has advantage on 5 years survival in patients with 1-4 mm depth of tumoral invasion. So radical vulvectomy and inguinofemoral lymph node disection should be preferred treatment approach in those cases. For tumors more than 4 mm depth of invasion, regional lymphadenectomy is a correct treatment choice due to risk of distant metastasis. Overall 5 year survival rate is 50%-60% for cases with MM. Altough new treatment modalities have been introduced, survival is nearly same within last 40 years^(10)^. Among patients with malign melanoma, 3 of them hadtumoral invasion depth between 1-2 mm underwent radical surgery. Two of these three patients had lymph node metastasis. In these three patients with MM, recurrence developed during follow-up. And recurrence was completely resolved after treatment and the patients were alive at the end of the follow-up period.

Basal cell cancer, which is seen in 2% of all vulvar cancers has generally good prognosis. However, if left untreated it can be locally destructive. Generally, local excisionis the preferred treatment^(11)^. However, lymphnode positivity or hematogenous metastasis has been reported in basal cell cancers in the literature^(12)^. Nearly 10%-20% of cases recur locally^(13)^. In our study, two offive patients diagnosed with basal cell cancer underwent radical surgery, local excision was performed for other three cases. In one case with basal cell carcinoma, vaginal recurrence occurred after 30 months of follow-up period. Chemotherapy (BOMP) was given for treatment of recurrence and there was complete clinical response. After 60-72 months of follow-up period, all five patients were alive with no disease.

In our study, mean duration of hospitalization was 15 days, it is similar to study of Brinton et al.^(14)^. The most common reported complication sofradical surgery are the surgical site infection and wound disruption, more rarely leg edema and lymphocyst^(15)^. In this study two patients had early post-op complications (one withsurgical site infection and another with wound disruption in the groin).

Non-squamous cell vulva cancers are heterogenous group of tumor and seen in extremely rare. Malign melanomais the most aggressive one in which distant metastasisare frequently encountered. The only known reality is that the prognosis of malign melanoma is better when only diagnosed at earlier stages. Forthe treatment of vulvar malignant melanomas, there is no consensus because there is small number of case series in the literature. Contribution of current treatment on survivalis also limited for malign melanoma. In order to make early diagnosis of non-squamous vulvar cancers and increase the survival, multi center studies with large group of patients are necessary.

## Figures and Tables

**Table 1:**
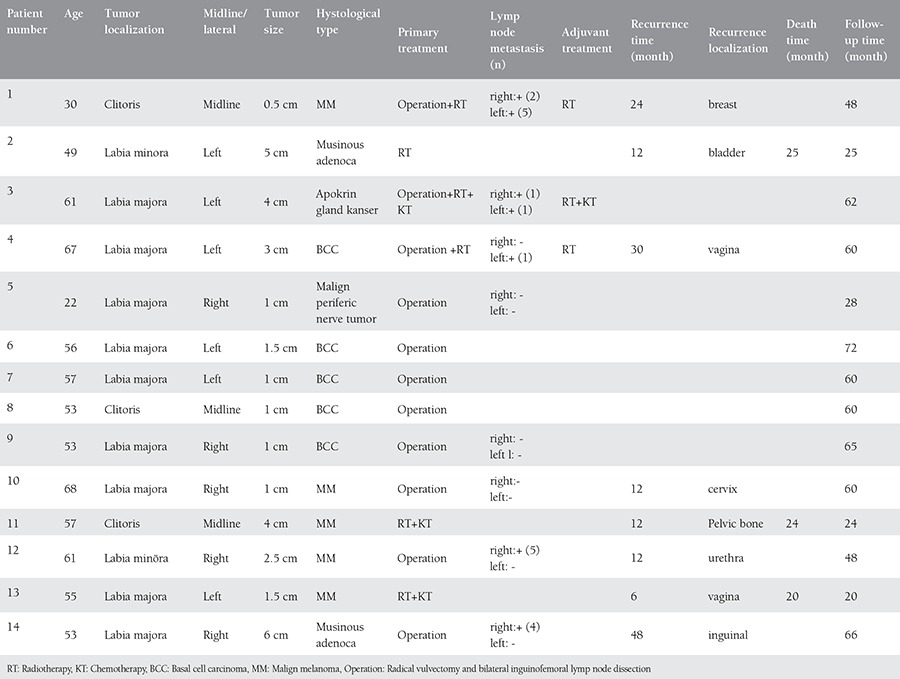
Tumor characteristics, treatment and follow-up schemes of patients with non-squamous vulvar cancer
